# “Let’s get back to normal”: emotions mediate the effects of persuasive messages on willingness to vaccinate for COVID-19

**DOI:** 10.3389/fpubh.2024.1377973

**Published:** 2024-05-02

**Authors:** Krista R. Muis, Panayiota Kendeou, Martina Kohatsu, Shuting Wang

**Affiliations:** ^1^Department of Educational and Counselling Psychology, McGill University, Montreal, QC, Canada; ^2^Department of Educational Psychology, University of Minnesota Twin Cities, St. Paul, MN, United States

**Keywords:** COVID-19 vaccine, vaccine hesitancy, persuasion, public education messaging, emotions

## Abstract

**Objective:**

We examined the effectiveness of three different messages for persuading individuals to get vaccinated against COVID-19, and the role that emotions play in persuasion.

**Methods:**

Four hundred-thirty-six participants reported their concern about the COVID-19 pandemic and confidence/hesitancy toward vaccines. Participants were randomly assigned to one of three text conditions: (1) self-interest: a persuasive message that focused on how much of a “serious threat COVID-19 is to you,” and to get vaccinated to “protect yourself”; (2) self-interest + altruistic: a persuasive message that focused on the “threat to you and your community” and to get vaccinated to “protect you and your loved ones”; (3) self-interest + altruistic + normal: a persuasive message that included (2) but added “This is the only way we can get back to a normal life.”; and, (4) a baseline control: no text. After reading, participants reported their emotions toward COVID-19 vaccines and their willingness to get vaccinated.

**Results:**

Individuals in the self-interest + altruistic + normal condition were more willing to get vaccinated compared to the control condition and self-interest + altruistic condition. However, there were no differences in willingness between the self-interest + altruistic + normal condition and the self-interest condition. Moreover, emotions mediated relations between vaccine confidence/hesitancy and willingness.

**Conclusion:**

A message that focuses on “getting back to normal” can achieve important public health action by increasing vaccine uptake to protect the population. Future work is needed across multiple countries and contexts (i.e., non-pandemic) to assess message effectiveness.

## Introduction

At the start of the COVID-19 pandemic, governments worldwide implemented extreme social distancing and quarantine measures to protect the most vulnerable and to help manage healthcare service demand. The need for a vaccine to protect the world population from the deadly virus took center stage, and so too did the anti-vaccination movement (1). Vaccines are an ideal public health strategy to prevent disease but vaccine hesitancy has become problematic due to numerous factors including complacency, inconvenience, lack of confidence, and mistrust in authorities (2). Vaccine hesitancy or resistance is defined as a position wherein an individual is uncertain about taking a vaccine or is completely against taking a vaccine, whereas vaccine acceptance is defined as the position wherein an individual accepts vaccines or actively demands them (3).

One method by which health authorities have attempted to combat vaccine hesitancy is through social persuasion (4). Social persuasion includes prompting, compelling, or inducing a change in people’s beliefs, understanding, behaviors, attitudes, or reactions toward something (5). Research has shown that positive messages designed to increase perceived value, importance, and effectiveness for engaging in social measures are better at changing people’s perceptions and prompting them to take action compared to neutral or negative messages (6, 7). Research has also shown how effective persuasive messages are in changing perceptions and behavioral intentions [e.g., (8, 9)]. Of particular relevance, in the context of COVID-19, research has shown that public education health messages that focus on both public and personal benefits (“protect yourself, protect others”) are more effective than addressing personal benefits alone (“protect yourself”) in increasing behavioral intentions like social distancing and wearing masks (10–12).

Indeed, several studies have been conducted to examine COVID-19 vaccine hesitancy in terms of worldwide rates of hesitancy, personal characteristics of hesitant individuals, and reasons for being hesitant [e.g., (3, 13–15)]. For example, drawing from large samples in Ireland and the United Kingdom (UK), Murphy et al. (3) found that 35 and 31%, respectively, of these samples were vaccine hesitant/resistant. For personal characteristics, they found that women and individuals in lower income brackets were more hesitant/resistant. For psychological variables, they found that vaccine hesitant/resistant individuals espoused a lower level of trust in health care professionals, scientists, and the government; had more negative attitudes toward immigrants, lower levels of altruism, stronger religious beliefs, were less agreeable, held higher levels of conspiratorial beliefs and higher internal locus of control. Finally, they found that individuals who were more hesitant/resistant were less likely to get information about COVID-19 from television, radio newspapers, and government agencies, but were more likely to obtain information from social media.

Head et al. (14) reported similar results with regards to vaccine intention whereby individuals who espoused liberal political views and altruistic beliefs had greater intentions to get vaccinated whereas individuals who were less educated were less likely to get vaccinated. Interestingly, intention to vaccinate significantly increased when individuals were told that health experts strongly recommended getting vaccinated against COVID-19. Although several studies have explored causal mechanisms for changing perceptions and behavioral intentions, relatively few have examined the role that emotions play in social persuasion, which is considered a key factor in social persuasion (6, 7, 16–18). Theoretically, it is essential to understand what factors facilitate or constrain social persuasion to ensure that messages are designed that effectively prompt individuals to get vaccinated. Accordingly, the purpose of this research was to explore the role of emotions on the effects of persuasive messages in increasing vaccine intentions specific to COVID-19 and to explore the effects of different kinds of messages on social persuasion more broadly.

### Social persuasion

Governments and individuals have engaged in social influence through persuasion as a widespread civil means of social control (4). Persuasion uses reason and emotion to sway individuals to change their attitudes and behaviors (5). Theorists agree that individual characteristics (e.g., personal relevance, motivation) and message characteristics (e.g., source credibility, argument structure) play significant roles in persuasion (19, 20). Specifically, as Petty and Cacioppo (20) argued in their Elaboration Likelihood Model (ELM), it is imperative to engage individuals in deeper processing of the message for long-lasting change to occur (21). To promote deeper processing, researchers have developed persuasive messages (22), which are designed to challenge individuals’ beliefs and provide them with new information. In the context of a COVID-19 vaccine, a persuasive message may be a text that challenges individuals’ beliefs about the seriousness of the pandemic and the importance of getting vaccinated. Highly persuasive messages must provide ample evidence to support the arguments raised, come from credible sources like experts, use powerful language (23), and draw an emotional response from readers (22).

Messages that are more positive (8, 9, 24), include more factual information (8, 9, 25, 26), and refer to sources that are more credible are more likely to increase willingness to change behaviors, and actually change those behaviors, compared to messages that include a more negative tone, less factual information, and less credible sources. Additionally, evidence from research on disease prevention has shown that messages that target both self-interested (i.e., protect yourself) and altruistic (i.e., protect others) motivations are effective in motivating individuals to vaccinate (27, 28). Why might this be the case? Emotions may play a role in social persuasion.

### The role of emotions in social persuasion

Emotions play an important role in individuals’ learning, motivation, attitudes, and performance (29, 30). Emotions are defined as multifaceted phenomena that consist of affective, cognitive, motivational, physiological, and expressive components (31). For example, anxiety that an individual has about vaccines may consist of feelings of uneasiness (affective), worry about allergic reaction to a vaccine (cognitive), need to avoid vaccines (motivation), sweaty palms and increased heart rate (physiological), and tense facial expression [expressive; (32)]. Emotions can also be described according to their valence (positive versus negative), arousal (activating versus deactivating), and object focus (e.g., social emotions, topic emotions, epistemic emotions, achievement emotions).

Research has shown that positive emotions, like joy, may increase effortful processing of information (33) whereas negative emotions, like frustration, may reduce effortful processing of information given that negative emotions can draw attentional resources away from the task an individual may be engaged in (34). When it comes to processing of textual information specifically, Bohn-Gettler (35) proposed that emotions influence reading comprehension wherein positive emotions, like hope, result in an increase of assimilative processing, like elaboration, to integrate new information into existing knowledge structures. However, when individuals experience higher positive emotions, they may ignore information that is inconsistent with their beliefs, which decreases the likelihood of individuals changing their beliefs when they are challenged (36, 37). This suggests that context needs to be taken into consideration as positive emotions do not always result in improved processing of information.

Moreover, when information is inconsistent with beliefs, this can trigger threat appraisals that prompt intense negative emotions like fear, anxiety, and anger (38). When this occurs, individuals may want to protect their beliefs and avoid negative emotions and, as such, ignore belief-inconsistent information. This may result in individuals learning less from those texts, if at all, particularly about controversial topics (36). However, negative emotions can also prompt accommodative processing (35). Specifically, information that is inconsistent with beliefs may trigger surprise (a neutral emotion) (39), followed by confusion, frustration, or anxiety (40). An individual may interpret these negative emotions as indicating that something is not quite right (40). When this occurs, individuals may engage in accommodation of belief structures or existing knowledge so that new information can be better incorporated.

In the context of COVID-19, researchers around the globe reported that individuals felt anger toward the lockdowns and removal of freedoms, and sadness about the number of people who died (41). Moreover, the World Health Organization (2) indicated that pandemic fatigue had become a major concern for adherence to the measures that were put in place to slow the spread of the virus. Given pandemic fatigue and the negative emotions associated with the pandemic, it may be necessary to create a persuasive message that addresses positive and negative emotions simultaneously so that individuals will process the information and change their beliefs about vaccines to protect themselves (self-interest) and others (altruistic). Moreover, to address pandemic fatigue, it may be the case that messages that focus on “getting back to normal” in addition to protecting oneself and others may be the most effective means by which to encourage individuals to engage in vaccine uptake. Arguably, if individuals can be persuaded that engaging in vaccine uptake will allow the world to get “back to normal,” this may increase joy, hope, and relief, and reduce anger.

To date, limited research has been done on the role of emotions in social persuasion (7), but some research has explored emotions in the context of the COVID-19 pandemic. To illustrate, Heffner et al. (42) examined positive versus negative emotions and willingness to engage in social isolation via a threatening (e.g., millions will die) or altruistic text (e.g., “save millions of lives”). They found that both texts increased willingness to isolate, but that the threatening text was highly arousing and moderately unpleasant whereas the altruistic text was moderately arousing and fairly pleasant. In another study, Pfattcheicher et al. (18) reported that empathy predicted willingness to engage in social distancing and wearing a face mask, which were key social distancing and protective measures against contracting COVID-19.

More research is needed to better understand whether and how emotions facilitate or constrain social persuasion and what types of messages are more effective than others in persuading individuals to get vaccinated. Fostering joy, hope, relief, and empathy and decreasing anger may be necessary to increase intentions to get vaccinated, particularly those who are vaccine hesitant or resistant. More importantly, individuals who are vaccine hesitant or resistant may need to be persuaded that vaccination is the only way to “get back to normal.” To date, although studies have been conducted to explore the efficacy of persuasive messages to get vaccinated [see (43–45)], our insight on the role of emotionally-driven persuasive messages on vaccine hesitancy remains limited. As such, the goal of this research was to develop a powerful, credible message to persuade individuals to get vaccinated for COVID-19 by reducing negative emotions and increasing positive emotions to foster more elaborative processing of the message.

We developed three different persuasive messages to evaluate whether they differed in their effectiveness on social persuasion, whether there were differences in emotions about COVID-19 after reading these messages, and whether emotions mediated relations between vaccine confidence and hesitancy and willingness to get vaccinated. The first text focused on protecting oneself from COVID-19 (self-interest condition), the second text focused on protecting oneself and others (self-interest + altruistic condition), and the third text focused on protecting oneself, others, and getting back to normal (self-interest + altruistic + normal). Given that level of concern about the pandemic predicts willingness to engage in preventive measures (12), we included concern about the pandemic as a covariate, and included a control condition wherein no textual/persuasive information was provided. Our research questions were as follows: (1) Are there differences in willingness to get vaccinated as a function of text condition (i.e., self-interest; self-interest + altruistic; self-interest + altruistic + let’s get back to normal) and vaccine hesitancy? (2) Are there differences in reported emotions as a function of text condition and vaccine hesitancy? (3) What is the relation between vaccine hesitancy and confidence, emotions, and willingness to get vaccinated?

Based on previous theoretical and empirical work (4, 18, 20, 43–45), we hypothesized that individuals in the self-interest + altruistic + normal persuasive text condition would report the highest willingness to get vaccinated compared to individuals in the other three conditions, with the control condition reporting the lowest level of willingness to get vaccinated (Hypothesis 1). We also predicted that individuals in the other two text conditions (self-interest, and self-interest + altruistic) would be more willing to get vaccinated than the control group, with no differences between the two text conditions (Hypothesis 2). We further predicted that individuals who were vaccine confident would be more willing to get vaccinated than those who were vaccine hesitant/resistant (Hypothesis 3).

**Figure 1:**
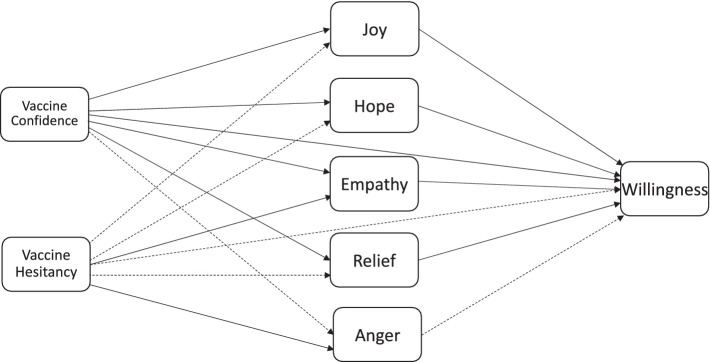
Hypothesized model. Dotted lines indicate negative relations.

For emotions, we hypothesized that individuals in the self-interest + altruistic + normal condition would report the highest level of joy, hope, and relief, and the lowest level of anger compared to the other three groups, with the other two text conditions reporting less anger and more joy, hope, empathy, and relief than the control condition (Hypothesis 4). We also hypothesized that vaccine hesitant individuals would report lower levels of joy, hope, and relief, but higher levels of anger about the COVID-19 vaccine compared to individuals who were vaccine confident (Hypothesis 5). We further hypothesized that vaccine confidence would positively predict joy, hope, empathy, and relief and negatively predict anger, whereas vaccine hesitancy would positively predict anger and empathy but negatively predict joy, hope, and relief (Hypothesis 6). We also predicted that anger would negatively predict willingness to get vaccinated whereas joy, hope, relief, and empathy would positively predict willingness to get vaccinated (Hypothesis 7). Finally, we hypothesized that emotions would mediate relations between vaccine confidence and hesitancy and willingness to get vaccinated (Hypothesis 8). The hypothesized model is presented in Figure 1.

## Method

### Participants

We conducted a power analysis using G*Power [Version 3.1; (46)]. Given previous research on social persuasion and health behaviors [e.g., Jordan et al. (11)], we expected small to medium effect sizes. With alpha set at 0.05 and power set at 0.80, power analysis revealed a required total sample size of 341. One hundred sixty-three participants were then recruited across Canada on April 12, 2021, using Amazon’s Mechanical Turk (MTurk). Another 285 participants from Canada were sampled by using a snowball sampling technique through Facebook. These two platforms were used for recruitment to ensure a more representative sample of individuals given that MTurk workers tend to be better educated, younger, and not racially diverse (47), whereas Facebook users range in age, educational level, race, and remain bipartisan in their news channels and political affiliations (48).

Of the 163 participants sampled using MTurk, five failed both attention checks and were subsequently removed. Five completed the survey in less than one minute (e.g., 11 s) and were also subsequently removed from the sample, for a sample of 153 participants from MTurk. Of the 285 participants sampled through Facebook, all but two completed the survey and passed both attention checks. The combined samples resulted in a total sample of 436 (*n* = 236 female, 155 male, 3 non-binary, 42 chose not to report). However, of the entire sample of 436, 98 reported having already received at least one dose of one of the COVID-19 vaccines available in Canada. As such, these individuals were removed from analyses since they were already vaccinated and did not need to be persuaded and another 12 were removed as they had missing data. The final analytic sample was 325. The average age was 37.81 years (range 17 to 75; *SD* = 14.11), with 78.1% reporting English as their first language, 52% reporting receiving a bachelor’s degree or higher, and 50% reporting a personal annual income of $65,000 CAD per year or less (14% chose not to answer). Except for Nunavut/Northwest Territories and the Yukon, all other provinces were represented.

### Materials

#### Concern about COVID-19

Seven self-report items were used to measure participants’ concern about the pandemic. Items were drawn from health-based research that assesses individuals’ perceived seriousness of an event; in this context, the negative consequences related to getting COVID (49). Previous research has shown that concern predicts the likelihood that individuals will take action to prevent illness or disease [see (50)]. Example items included, “How concerned are you at present about the coronavirus pandemic?” and “In terms of the pandemic, how concerned are you about your own physical health?” Participants rated each item on a 5-point Likert scale with anchors for each value: 1 “Not at all,” 2 “A little,” “Moderately,” “Very much,” and 5 “Extremely” concerned. Cronbach’s alpha reliability estimate was good at α = 0.73.

#### Vaccine confidence and hesitancy

A sixteen-item self-report scale was used to measure participants’ confidence in vaccines and their hesitancy toward them. Items were drawn from a previously validated instrument (51). Nine items measured participants’ confidence in vaccines (e.g., “Vaccines are effective in preventing diseases”), and the remaining seven items measured participants’ hesitancy toward vaccines (e.g., “Vaccines have negative side effects that outweigh the benefits of vaccination”). Participants were instructed to rate the degree to which they agreed with each statement on vaccinations, using a rating scale from 1 “Strongly Disagree” to 5 “Strongly Agree,” with 3 “Neither Agree nor Disagree” as the middle option. Items for each subscale were summed and averaged, with higher scores reflecting more vaccine confidence for the one subscale, and more vaccine hesitancy for the other subscale. Reliability for each subscale was good; Cronbach’s alpha for the vaccine confidence subscale was 0.86, and 0.73 for the vaccine hesitancy subscale.

#### Experimental texts

Three experimental texts were developed based on content from the Centers for Disease Control website on COVID-19 vaccinations.^1^ Except for the personal message component, all features of the texts were identical (e.g., used persuasive language, the same credible sources, and written to be personally relevant). The first 68 words were the same across all three texts, which began by providing basic information about COVID-19 and stating how contagious COVID-19 is. The texts then described it as a serious threat and recommended that the threat should be taken seriously to prevent further spread. The texts then presented information with regards to vaccines, their safety and efficacy, and then provided encouragement to get vaccinated.

The key differences between the three texts were minor wording that focused on protecting oneself (self-interest), protecting oneself and others (self-interest + altruistic), or protecting oneself and others as well as getting back to a normal life (self-interest + altruistic + normal). For example, for the text that focused on protecting oneself, following the information on how contagious the virus is, the text stated, “This means COVID-19 is a *serious threat to you*,” whereas the other two texts stated, “This means COVID-19 is a *serious threat to you and your community*.” As another example, the text that focused on personal protection stated, “COVID-19 vaccination helps *protect you* from getting sick or severely ill with COVID-19” whereas the other two texts stated, “COVID-19 vaccination will help *protect you* from getting sick or severely ill with COVID-19 and will help *protect your loved ones* and the people around you. That is, even if you do get COVID-19 after being vaccinated, it may also prevent you from spreading it to others.” Finally, for the text that focused on getting back to normal, the text added the following, “People who have been fully vaccinated can start to do some things that they had stopped doing because of the pandemic. Countries like the UK and Israel are getting back to their normal life because everyone is doing their part and getting vaccinated. To stop this pandemic, everyone will need to get vaccinated. This is the only way we will be able to *get back to a normal life*. *Protect yourself and others* from COVID-19. Let’s get back to normal!” All texts then ended with “*Do not wait. Vaccinate!*” and were followed by a pamphlet that highlighted the main message (i.e., protect oneself; protect oneself and others; protect oneself and others, and let’s get back to normal). See Appendix A for the texts and pamphlets. Total word count for the texts were 172, 224, and 298, respectively, with a Flesch reading ease score of 46.3, and a Flesch–Kincaid grade level of 9.9 for all three texts.

#### Emotions

A self-report questionnaire consisting of five items was used to measure participants’ emotions toward COVID-19 vaccines. Each item consisted of a single word (e.g., “Happy”) and participants were asked to report the intensity of their emotional response to COVID-19 vaccines (control condition) after they read the text (text conditions). Single-item measures have demonstrated to be psychometrically sound substitutes for multi-item scales when administration time is short [e.g., (52)]. Intensity was reported using a 5-point Likert scale using the following labels: Not at all (1), Very little (2), Moderate (3), Strong (4), and Very Strong (5). The five emotions included: joy, hope, empathy, relief, and anger.

#### Willingness to get a COVID-19 vaccine

A seven-item measure was developed to assess participants’ willingness to get a COVID-19 vaccine (e.g., “In light of the COVID-19 outbreak, I am willing to…”). Participants were asked to rate their willingness using a sliding rating scale that ranged from 0 “Not at all willing to do this” to 100 “Very willing to do this,” with 50 “Moderately willing to do this” as the middle marker. The first item assessed general willingness to get a COVID-19 vaccine (e.g., “Get a COVID-19 vaccine”), along with more specific options including choice of vaccine (e.g., “Get a COVID-19 vaccine if I can choose which one I get”), no choice (e.g., “Get a COVID-19 vaccine even if I cannot choose which one I get”), and then willingness to get a specific vaccine currently available in Canada (e.g., “Get the Pfizer/Moderna/AstraZeneca/Johnson and Johnson vaccine for COVID-19”). Chronbach’s alpha reliability for the seven-item scale was 0.90.

#### Demographic information

Participants reported their age, sex, first language spoken, highest level of education completed, current health status (ranging from poor to excellent), what health issues they have, how frequently they get the flu vaccine, current employment status, essential worker status, marital status, annual income, residence location (e.g., postal code; urban, suburban, or rural area, etc), number of parents/children/individuals living with them, political affiliation, strength of political affiliation for social issues, strength of political affiliation for economic issues, time spent per day following information about COVID-19, sources of that information (e.g., CBC, Facebook, Fox News, Radio-Canada, CNN), and religiosity (e.g., religious affiliation and strength of beliefs).

### Procedure

After obtaining ethics approval from the Research Ethics Board (REB), participants were recruited through MTurk and via a snowball sampling technique through social media (Facebook). A link was provided to Qualtrics (on MTurk and Facebook, housed by our university to ensure encrypted procedures were strictly followed), which was the platform used for collecting data. Participants names were not collected to ensure anonymity and all information was stored on a secure, locked computer with double authentication measures to ensure confidentiality. Only the first author had access to the data, which was all in numerical form. Participants first consented, after which they completed the vaccine confidence and hesitancy questionnaire. Participants were then randomly assigned to one of four conditions: protect yourself “self-interest” condition; protect yourself and others “self-interest + altruistic” condition; protect yourself, others, and let’s get back to normal “self-interest + altruistic + normal” condition; or the control condition (no persuasive message).

After reading (or not in the case of the control condition), participants reported their emotions about COVID-19 vaccines followed by their willingness to get a COVID-19 vaccine. Participants then completed the demographics questionnaire after which they were paid for their time (MTurk) or were entered into a draw to win $100 (with a chance of winning being 1 in 50). To be entered into the draw, participants recruited through Facebook were provided the first author’s email and were asked to contact the first author with a randomly generated code provided at the end of the survey. Participants in the control condition spent approximately 10 min completing the survey, whereas participants in the text conditions spent approximately 12 min (self-interest) to 13 min (self-interest + altruistic, and self-interest + altruistic + normal) completing the survey and reading the texts.

## Results

### Preliminary data screening and analyses

Prior to conducting analyses, it was first necessary to check for normality and outliers, and whether groups differed on concern, vaccine confidence, and vaccine hesitancy. As expected, most variables were skewed due to the nature of the items, which is common in research on vaccine hesitancy and in research on emotions [see (32)]. Moreover, as expected, there were no differences between groups on concern about COVID-19, *F*(3, 325) = 1.08, *p* > 0.05, vaccine confidence, *F*(3, 325) = 2.19, *p* > 0.05, or vaccine hesitancy, *F*(3, 325) = 1.23, *p* > 0.05. Table 1 reports means and standard deviations for willingness to get vaccinated and emotions as a function of condition, and Table 2 reports correlations between all variables.

**Table 1:** Means and standard deviations for willingness to get vaccinated and emotions as a function of text condition and hesitancy.

	Control (*n* = 86)	Self-interest (*n* = 76)	Self-interest + Altruistic (*n* = 84)	Personal + Altruistic + Normal (*n* = 79)
Vaccine confident				
Willingness	75.94 (23.92)	85.43 (14.88)	75.20 (25.46)	84.45 (20.80)
Joy	3.32 (1.18)	3.95 (0.97)	3.72 (1.08)	4.22 (0.89)
Hope	3.61 (1.07)	4.24 (0.82)	4.05 (0.93)	4.25 (0.84)
Empathy	2.75 (1.26)	3.13 (1.29)	3.00 (1.26)	2.84 (1.40)
Relief	3.27 (1.21)	3.93 (0.99)	3.67 (1.10)	3.87 (1.02)
Anger	1.85 (1.03)	1.50 (0.94)	1.50 (0.89)	1.65 (0.88)
Vaccine hesitant/Resistant				
Willingness	52.93 (29.09)	49.14 (28.92)	47.39 (35.22)	63.64 (25.52)
Joy	2.67 (1.24)	2.60 (0.99)	2.88 (1.50)	3.69 (1.08)
Hope	3.04 (1.16)	3.13 (1.06)	3.05 (1.43)	3.75 (1.00)
Empathy	2.70 (1.45)	2.40 (1.18)	2.94 (1.47)	2.43 (1.50)
Relief	2.62 (1.13)	2.73 (0.96)	2.94 (1.29)	3.43 (0.96)
Anger	2.29 (1.26)	2.46 (1.18)	2.29 (1.35)	2.18 (1.10)

Standard deviation is in (brackets).

**Table 2:** Zero-order correlations between variables.

	Hesitancy	Joy	Hope	Empathy	Relief	Anger	Willingness
Confidence	−0.500**	0.56**	0.567**	0.306**	0.603**	−0.277**	0.625**
Hesitancy		−0.46**	−0.427**	−0.111*	−0.469**	0.366**	−0.501**
Joy			0.70**	0.26**	0.74**	−0.45**	0.53**
Hope				0.403**	0.735**	−0.428**	0.493**
Empathy					0.277**	−0.065	0.222**
Relief						−0.406**	0.512**
Anger							−0.254**

^**^Significant at *p* < 0.001. ^*^Significant at *p* < 0.05.

### Sample characteristics for vaccine hesitancy

To assess whether our sample of vaccine hesitant/resistant individuals was consistent with previous literature (3, 13–15), we first identified whether individuals were hesitant/resistant or not. Based on participants’ score on vaccine hesitancy (i.e., an average score higher than 3, the neutral point on the scale), participants were coded as vaccine hesitant/resistant (23%) or vaccine confident (77%). Consistent with previous research, individuals who were vaccine hesitant/resistant were primarily female (62%), Catholic (25%), moderately to extremely religious (47%), but also identified mostly to the Liberal Party of Canada (46%).

Interestingly, there were no differences in vaccine hesitancy between those sampled from MTurk and those from Facebook (χ^2^ = 2.94, *df* = 1, *p* = 0.09) and individuals who were vaccine hesitant did not differ in their information sources for COVID-19 from those who were vaccine confident. For both groups, 22% obtained their information from CBC News (the most frequent source). Moreover, regression analyses revealed that vaccine hesitancy was not predicted by age (*p* = 0.62), health status (ranging from poor to excellent; *p* = 0.72), number of health issues (*p* = 0.96), level of concern about the pandemic (*p* = 0.21), or level of education (*p* = 0.40). However, level of religiosity was a significant positive predictor wherein stronger religious beliefs predicted more hesitancy, *β* = −0.27, *p* < 0.001.

### Effect of persuasive messages on willingness to get vaccinated

To examine the first research question, whether groups differed on willingness to get vaccinated as a function of type of persuasive message and hesitancy, using concern as a covariate, ANCOVA results revealed a main effect of text condition, *F*(3, 325) = 2.80, *p* = 0.04, *η^2^* = 0.03, a main effect of hesitancy group, *F*(1, 325) = 70.54, *p* < 0.001, *η^2^* = 0.18, but no interaction, *F*(3, 325) = 1.07, *p* > 0.05. As hypothesized, individuals who were confident in vaccines were more willing to get vaccinated than those who were hesitant/resistant (Hypothesis 3).

Follow-up *post hoc* analyses using LSD revealed that individuals in the self-interest + altruistic + normal condition were more willing to get vaccinated compared to the control condition (*p* = 0.004) and self-interest + altruistic condition (*p* = 0.004) (Hypothesis 1). However, counter to our hypothesis, there were no differences in willingness between the self-interest + altruistic + normal condition and the self-interest condition. Similarly, individuals in the self-interest condition were more willing to get vaccinated compared to individuals in the self-interest + altruistic condition (*p* = 0.02) and control condition (*p* = 0.02) (Hypothesis 2). Finally, counter to our hypothesis, individuals in the self-interest + altruistic condition did not differ on willingness compared to the control condition (*p* > 0.05).

We further explored whether vaccine type mattered, and whether choice or no choice as to which vaccine individuals received mattered with regard to messaging and willingness. Indeed, despite large differences in willingness across the various vaccines (particularly high willingness for Pfizer and Moderna, but low for Astrazeneca and Johnson and Johnson), the same patterns of results were replicated, with much higher willingness to get vaccinated (upward of 20%) when individuals were given the choice of which vaccine to receive compared to when they were not given a choice.

### Effect of persuasive texts on emotions

For the second research question, whether emotions differed as a function of text condition and hesitancy, for joy, ANCOVA results revealed a significant main effect of text condition, *F*(3, 325) = 7.92, *p* < 0.001, *η^2^* = 0.07, a main effect of hesitancy group, *F*(1, 325) = 33.23, *p* < 0.001, *η^2^* = 0.10, but no interaction, *F*(3, 325) = 1.63, *p* > 0.05. As hypothesized, individuals who were vaccine confident expressed more joy in relation to the COVID-19 vaccine compared to individuals who were vaccine hesitant/resistant (Hypothesis 5). *Post hoc* follow-up analyses using LSD revealed that, as hypothesized, individuals in the self-interest + altruistic + normal condition expressed significantly more joy about the COVID-19 vaccine compared to individuals in the other three conditions (all *p* < 0.01). Individuals in the self-interest and self-interest + altruistic conditions also expressed significantly greater joy than those in the control condition (both *p*s < 0.01), and no differences were found in joy between individuals in the self-interested condition and the self-interested + altruistic condition (Hypothesis 4).

For hope, ANCOVA results revealed a significant main effect of text condition, *F*(3, 325) = 4.73, *p* = 0.003, *η^2^* = 0.04, a main effect of hesitancy group, *F*(1, 325) = 35.67, *p* < 0.001, *η^2^* = 0.10, but no interaction, *F*(3, 325) = 1.27, *p* > 0.05. As hypothesized, individuals who were vaccine hesitant/resistant were less hopeful about the vaccine than those who were vaccine confident. *Post hoc* follow-up analyses using LSD revealed that there were no differences between the three persuasive text conditions on hope, but that individuals in all text conditions were significantly more hopeful than individuals in the control condition (all *p* < 0.001).

For empathy, ANCOVA results revealed no significant effects or interactions (all *p* > 0.05). For relief, ANCOVA results revealed a significant main effect of text condition, *F*(3, 325) = 4.16, *p* = 0.007, *η^2^* = 0.04, a main effect of hesitancy group, *F*(1, 325) = 25.95, *p* < 0.001, *η^2^* = 0.08, but no interaction, *F*(3, 325) = 1.08, *p* > 0.05. As hypothesized, individuals who were vaccine hesitant/resistant were less relieved about the vaccine than those who were vaccine confident. *Post hoc* follow-up analyses using LSD revealed that there were no differences between the three persuasive text conditions on relief, but that individuals in all text conditions were significantly more relieved than individuals in the control condition (all *p* < 0.001). Finally, for anger, ANCOVA results revealed no main effect of text condition, *F*(3, 325) = 0.37, *p* > 0.05, but a main effect of hesitancy group, *F*(1, 325) = 24.63, *p* < 0.001, *η^2^* = 0.07, and no interaction, *F*(3, 325) = 0.76, *p* > 0.05. As hypothesized, individuals who were vaccine hesitant/resistant were more angry about the vaccine than those who were vaccine confident.

### Relations between vaccine confidence, hesitancy, emotions, and willingness to get vaccinated

**Figure 2:**
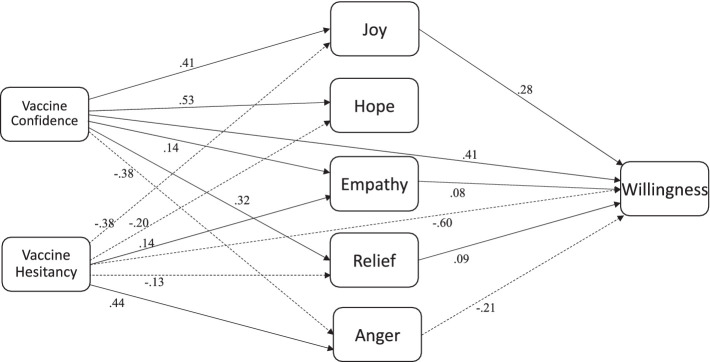
Final model.

To answer the last research question regarding relations between vaccine confidence, hesitancy, emotions, and willingness to get vaccinated, a path analysis using Mplus (53) was conducted (Figure 1). The model revealed an excellent fit, χ^2^ = 28.13, df = 3, *p* < 0.001, CFI = 0.96, RMSEA = 0.06 (Figure 2). Vaccine confidence negatively predicted anger (*β* = −0.38, *p* < 0.001) and positively predicted joy (*β* = 0.41, *p* < 0.001), hope (*β* = 0.53, *p* < 0.001), relief (*β* = 0.32, *p* < 0.001), *p* < 0.001, empathy (*β* = 0.14, *p* = 0.004) and willingness to get vaccinated (*β* = 0.59, *p* < 0.001). In contrast, vaccine hesitancy positively predicted anger (*β* = 0.44, *p* < 0.001) and empathy (*β* = 0.14, *p* < 0.001), but negatively predicted joy (*β* = −0.38, *p* < 0.001), hope (*β* = −0.20, *p* < 0.001), relief (*β* = −0.13, *p* = 0.002), and willingness to get vaccinated (*β* = −0.60, *p* < 0.001). Joy also positively predicted willingness to get vaccinated (*β* = 0.28, *p* < 0.01), as did relief (*β* = 0.09, *p* < 0.05), and empathy (*β* = 0.08, *p* < 0.05), whereas anger negatively predicted willingness to vaccinated (*β* = −0.21, *p* < 0.001). Mediation analyses further revealed that anger (−0.08, *p* < 0.001) and relief (0.03, *p* = 0.04) mediated relations between vaccine confidence and willingness, whereas anger (0.09, *p* < 0.001) and empathy (0.02, *p* = 0.04) mediated relations between vaccine hesitancy and willingness. We discuss these results next.

## Discussion

The purpose of this study was to examine the effects of three different types of persuasive messages on willingness to get vaccinated for COVID-19. We also explored the role that emotions play in social persuasion to better understand the mechanisms underlying social persuasion via text-based messages. Results revealed that consistent with hypotheses, the text that focused on getting back to normal in addition to protecting oneself and others (self-interest + altruistic + normal) was more effective in persuading individuals to get vaccinated compared to the control condition (no message) and the self-interest + altruistic condition. However, there were no differences in willingness to get vaccinated between the self-interest + altruistic + normal condition and the self-interest condition, and no differences between the control condition and the self-interest + altruistic condition.

The finding that the self-interest + altruistic condition did not affect willingness to get vaccinated is counter to recent research that found that self-interest + altruistic persuasive messages were more effective in increasing individuals’ behavioral intentions like social distancing/behaviors and wearing masks compared to no persuasive message (10–12). Arguably, such social behaviors may be construed as relatively easy to engage in to protect others compared to getting vaccinated, particularly for those individuals who are vaccine hesitant/resistant. As such, a message that focuses on protecting others may not be an effective way to encourage individuals to get vaccinated. However, the persuasive message about protecting oneself was just as effective in increasing individuals’ willingness to get vaccinated compared to the message that focused on getting back to normal, which also had an altruistic component to it, but only for individuals who were *not* vaccine resistant. To explain these results, we looked deeper into the effects of each of the messages as a function of individuals’ hesitancy toward vaccines.

### Vaccine hesitancy and persuasion

A close examination of the effects of each type of message as a function of vaccine hesitancy group (see Table 1) shows that for vaccine hesitant individuals, the *only* message that increased willingness to get vaccinated was “Let’s get back to normal.” Given that the other two persuasive text conditions had means lower than the control condition for individuals who were vaccine hesitant, and that the “normal” condition increased willingness by over 10% compared to the control condition, we interpret this result as meaningful and important from a public messaging perspective. In the context of pandemic fatigue (2), to persuade vaccine hesitant/resistant individuals to get vaccinated may require a focus on getting life back to normal with regards to the removal of restrictions and regaining of individual freedoms.

For vaccine confident individuals, both the self-interest condition and the self-interest + altruistic + normal condition increased willingness to get vaccinated by 10% above the control condition. These results suggest that in the context of a pandemic, individuals who are confident in vaccines are willing to protect themselves but are also wanting to get life back to normal. It may be the case that individuals were more driven to prevent themselves from getting seriously sick or dying than they were for protecting others from getting sick. Alternatively, at the time that vaccines were rolling out, it was not clear whether or to what extent the COVID-19 vaccines decreased viral load or spread of the virus and, as such, the message to protect others may not have been convincing to individuals since information was rapidly changing at that time (54). Taken together, these results suggest that context matters, and that messaging needs to be tailored as a function of individuals’ beliefs about vaccines and other psychological variables like choice versus no choice.

Indeed, a brief examination of the history of the anti-vaccination movement has shown that vaccine hesitancy has been around since the dawn of vaccines [see (55)]. Factors that affect vaccine hesitancy include complacency (perceived low risk, low general knowledge and awareness), confidence (trust in vaccine safety, the system or policy makers), convenience (availability, accessibility, affordability), calculation (engagement in gathering extensive information), and collective responsibility (willingness to protect others) (56). Historically, religious beliefs (i.e., “it is not God’s will”) and mandatory programs sparked a distrust in vaccines and riots due to restrictions on personal freedoms (57, 58). Modern-era distrust of vaccines grew from concerns over vaccine safety and efficacy, particularly after the polio vaccine was released with a live, active virus that had negative repercussions for a small proportion of children who were given the vaccine (59). Today, factors like religious beliefs, cultural beliefs, and perceptions of risk and harm continue to drive vaccine hesitancy. Prior vaccine history, perceived safety of vaccines, the impacts of vaccine mandates, political affiliation, information and misinformation on the internet, and satisfaction with government decision-making on other aspects of COVID-19 prevention or strategy management also played a significant role in the uptake of COVID-19 vaccines (60).

Results from our study provide further evidence of these factors playing a role. That is, individuals with strong religious beliefs were more vaccine hesitant and were more likely to affiliate with the liberal government of Canada, which is counter to what is typically found in the US with Republicans being more vaccine hesitant (61). What is particularly noteworthy with our results is that choice mattered for individuals, regardless of whether they were vaccine confident or hesitant. Indeed, willingness to get vaccinated was 90% for individuals in the “let’s get back to normal” and “personal” conditions but dropped to 72% for those same conditions when choice of vaccine was removed. These percentages dropped to 62 and 54%, respectively, when Astrazeneca was the option, reflecting individuals’ distrust in this vaccine given news of blood clots being a risk factor. Moreover, as previously noted, for vaccine hesitant individuals, willingness was significantly higher with the message of getting back to normal than any other message, and this was particularly pronounced when they had the choice of vaccines (82% willing) versus when they did not have a choice (46% willing). Accordingly, there may have been some additive effects for vaccine hesitant individuals where the message of getting back to normal coupled with a choice of vaccine was the most powerful approach to social persuasion. To further understand the mechanisms involved in social persuasion, it is also important to consider the role of emotions.

### The role of emotions in persuasion

Indeed, for all three persuasive message conditions, individuals felt more joy, hope, and relief than those in the control condition, with no differences in level of emotional intensity for the three persuasive message conditions (with the exception of joy). Moreover, no differences were found between persuasive text conditions and the control condition for anger or empathy. These results suggest that the persuasive messages had equal effects on increasing hope and relief, regardless of the type of persuasive message, and had no effect on empathy or anger. Most important, for the condition that included the message of getting back to normal, individuals expressed the greatest joy compared to individuals in the other three conditions.

As previous empirical work has demonstrated, positive emotions, like joy, can increase effortful processing of information (33) and result in assimilation of new information into current knowledge structures (35). As such, it appears that in this context, joy played a significant role in increasing individuals’ willingness to get vaccinated, perhaps from a belief that things will get back to normal. Indeed, results from path analyses revealed that greater vaccine confidence predicted more joy, relief, hope and empathy and less anger, whereas greater vaccine hesitancy negatively predicted joy, hope, and relief, but positively predicted anger and empathy. Moreover, the more angry individuals were about the vaccine, the less willing they were to get vaccinated. However, the more joy, hope, relief, and empathy they experienced, the more willing they were to get vaccinated. These emotions also mediated relations between vaccine confidence and hesitancy wherein for confidence, joy and relief were positive mediators whereas anger was a negative mediator and, for hesitancy, relief was a negative mediator whereas empathy was a positive mediator.

These results have important implications for the effects that emotions have on processing persuasive information, particularly when the message focuses on getting life back to normal under pandemic circumstances. Drawing from the emotions literature (32), it may be the case that processing of the persuasive messages was enhanced due to an increase in joy, hope, and relief across all three conditions. These results suggest that persuasive messages that focus on getting back to normal could persuade the largest number of individuals to get vaccinated, particularly those who are vaccine hesitant/resistant. Although vaccine campaigns have targeted vaccine safety and protection of oneself (self-interest) and others (altruistic), an additional focus on getting back to a normal life may be key to achieving a high vaccine uptake. In the context of COVID-19 where, in Canada, many restrictions were put into place that limited individuals’ freedoms (particularly in the province of Quebec), a focus on regaining those freedoms via vaccination and “getting back to normal” may have been a powerful approach to social persuasion. In other contexts, this “normal” message may not have been effective if freedoms were not restricted. As such, the efficacy of this approach may not translate to other situations where freedoms are not threatened.

### Implications, limitations, and future directions

Taken together, results from this study have broader vaccine education and promotion implications. Messages from trustworthy sources are important to incorporate into health promotion messaging, along with a highlight of the safety of the vaccine. Given the history of vaccine hesitancy (55), mandating vaccines is not a good choice to promote vaccine uptake. Rather, results from this study suggest that choice is critical as is a focus on freedoms rather than the removal of them. Education about the safety and efficacy of vaccines is also critical (55). But in the context of rapidly changing information about COVID-19 and vaccines, this element of vaccine safety and efficacy was nearly impossible, so freedom of choice may have been key. Results from this research also suggest that positive emotional appeals may prompt individuals to be less resistant to vaccines and foster confidence in their use.

From an information processing perspective (35), it may be the case that positive emotions foster a deeper processing of educational information about vaccines. Future research is needed to evaluate precisely how emotions impact information processing, particularly for vaccine hesitant individuals. For example, a think-emote-aloud protocol [see (62)] may be an effective way to capture individuals’ emotions and cognitive and metacognitive processes to examine their interplay during reading of persuasive messages, particularly for socio-scientific issues like vaccine hesitancy. Future research is also needed that takes into consideration other factors that affect vaccine uptake like perceived susceptibility, threat or severity of illness, and the potential role of community engagement. Moreover, our study was conducted in Canada, which, culturally, is considered a more socialist country compared to others like the U.S. What was surprising to us was the finding that “protecting others” did not have the positive effect on willingness as it has in the past in the Canadian context [see (12)]. Future work is needed to disentangle why this may have been the case and whether other cultures that are more or less collectivist or socialist would respond in similar ways to “getting back to normal.”

One limitation of this study is that we did not include altruistic only or normal only message conditions to better determine what specific aspect of the messages were most effective in increasing individuals’ willingness to get vaccinated. A second limitation of this study is that we did not measure actual vaccine uptake. Although vaccine intentions are a strong predictor of behavior (63), a more powerful evaluation of the effectiveness of our messages would have been to include a follow-up assessment as to whether individuals got vaccinated for COVID-19 or not. Future research should also consider interviewing individuals to better understand the effects of persuasive messages and why individuals were more willing (or not) to get vaccinated. A better understanding of the underlying mechanisms of persuasion will allow for improved persuasive messages that may more effectively combat vaccine hesitancy and resistance.

## Data availability statement

The raw data supporting the conclusions of this article will be made available by the authors, without undue reservation.

## Ethics statement

The studies involving humans were approved by Research Ethics Review Board at McGill University. The studies were conducted in accordance with the local legislation and institutional requirements. The participants provided their written informed consent to participate in this study.

## Author contributions

KM: Conceptualization, Data curation, Formal analysis, Funding acquisition, Investigation, Methodology, Project administration, Resources, Software, Supervision, Validation, Visualization, Writing – original draft, Writing – review & editing. PK: Conceptualization, Methodology, Validation, Visualization, Writing – review & editing. MK: Writing – review & editing. SW: Writing – review & editing.

## Footnotes

^1^ https://www.cdc.gov/coronavirus/2019-ncov/vaccines/index.html
