# Supplementary material for: “Let’s get back to normal”: emotions mediate the effects of persuasive messages on willingness to vaccinate for COVID-19
Source: Front Public Health. 2024 May 2;12:1377973. doi: 10.3389/fpubh.2024.1377973 (PMC11098132; doi:10.3389/fpubh.2024.1377973)

**Appendix**

**Self-Interest Text**

Please read the following information from Health Canada and the Centers for Disease

Control and Prevention about COVID-19, which the World Health Organization classified as a

pandemic on March 11, 2020.

Coronavirus disease 2019 (COVID-19) is a respiratory illness that can spread from person to

person. COVID-19 is very contagious. And people can spread COVID-19 before experiencing

any symptoms.

This means COVID-19 is a **serious threat to you**. It is recommended that you take this threat

very seriously to prevent contracting COVID-19.

Fortunately, **vaccines** are now available that can prevent you from getting COVID-19.

COVID-19 vaccines are **safe**. Over 170 million people in Canada and the United States have

received COVID-19 vaccines under the most intense safety monitoring in history.

All COVID-19 vaccines currently available in Canada are **highly effective at preventing**

COVID-19.

COVID-19 vaccination helps **protect you** from getting sick or severely ill with COVID-19.

Health Canada and the Centres for Disease Control recommend you get a COVID-19 vaccine

**as soon as you are eligible**.

**Protect yourself from COVID-19.**

**Don’t wait. Vaccinate!**

https://www.canada.ca/en/public-health/services/diseases/coronavirus-disease-covid-

19/vaccines.html

https://www.cdc.gov/coronavirus/2019-ncov/vaccines/vaccine-benefits.html

10


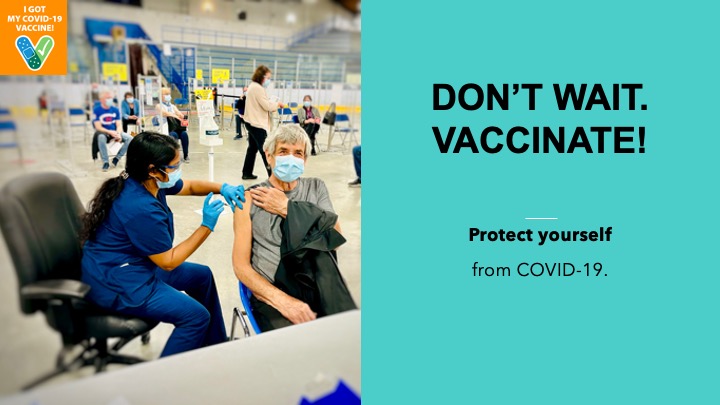


**Self-Interest plus Altruistic Text**

Please read the following information from Health Canada and the Centers for Disease

Control and Prevention about COVID-19, which the World Health Organization classified as a

pandemic on March 11, 2020.

Coronavirus disease 2019 (COVID-19) is a respiratory illness that can spread from person to

person. COVID-19 is very contagious. And people can spread COVID-19 before experiencing

any symptoms.

This means COVID-19 is a **serious threat to you and your community**. It is recommended

that you take this threat very seriously to prevent contracting COVID-19 and spreading it to

other people including your loved ones.

Fortunately, **vaccines** are now available that can prevent you and others from getting

COVID-19.

COVID-19 vaccines are **safe**. Over 170 million people in Canada and the United States have

received COVID-19 vaccines under the most intense safety monitoring in history.

All COVID-19 vaccines currently available in Canada are **highly effective at preventing**

COVID-19.

COVID-19 vaccination will help **protect you** from getting sick or severely ill with COVID-19

and will help **protect your loved ones** and the people around you. That is, even if you do get

COVID-19 after being vaccinated, it may also prevent you from spreading it to others.

Health Canada and the Centres for Disease Control recommend you get a COVID-19 vaccine

**as soon as you are eligible**.

**Protect yourself and others from COVID-19.**

**Don’t wait. Vaccinate!**

https://www.canada.ca/en/public-health/services/diseases/coronavirus-disease-covid-

19/vaccines.html

https://www.cdc.gov/coronavirus/2019-ncov/vaccines/vaccine-benefits.html

12


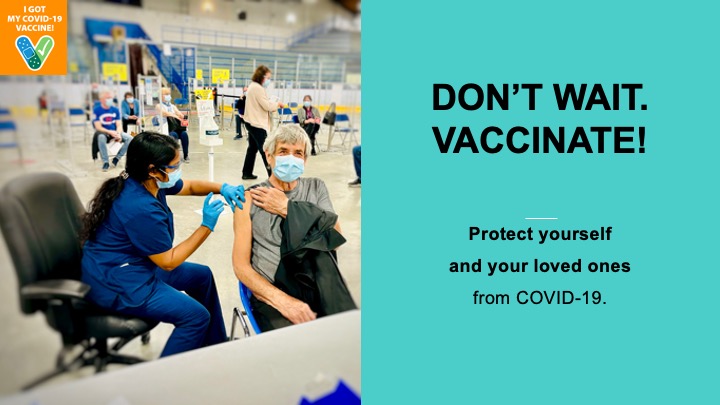


**Self-Interest plus Altruistic plus Normal Text**

Please read the following information from Health Canada and the Centers for Disease

Control and Prevention about COVID-19, which the World Health Organization classified as a

pandemic on March 11, 2020.

Coronavirus disease 2019 (COVID-19) is a respiratory illness that can spread from person to

person. COVID-19 is very contagious. And people can spread COVID-19 before experiencing

any symptoms.

This means COVID-19 is a **serious threat to you and your community**. It is recommended

that you take this threat very seriously to prevent contracting COVID-19 and spreading it to

other people including your loved ones.

Fortunately, **vaccines** are now available that can prevent you and others from getting

COVID-19.

COVID-19 vaccines are **safe**. Over 170 million people in Canada and the United States have

received COVID-19 vaccines under the most intense safety monitoring in history.

All COVID-19 vaccines currently available in Canada are **highly effective at preventing**

COVID-19.

COVID-19 vaccination will help protect you from getting sick or severely ill with COVID-19

and will help protect your loved ones and the people around you. That is, even if you do get

COVID-19 after being vaccinated, it may also prevent you from spreading it to others.

Health Canada and the Centres for Disease Control recommend you get a COVID-19 vaccine

**as soon as you are eligible**.

People who have been fully vaccinated can start to do some things that they had stopped

doing because of the pandemic. Countries like the UK and Israel are getting back to their

normal life because everyone is doing their part and getting vaccinated.

To stop this pandemic, everyone will need to get vaccinated. This is the only way we will be

able to get back to a normal life.

**Protect yourself and others from COVID-19. Let’s get back to normal!**

**Don’t wait. Vaccinate!**

https://www.canada.ca/en/public-health/services/diseases/coronavirus-disease-covid-

19/vaccines.html

https://www.cdc.gov/coronavirus/2019-ncov/vaccines/vaccine-benefits.html


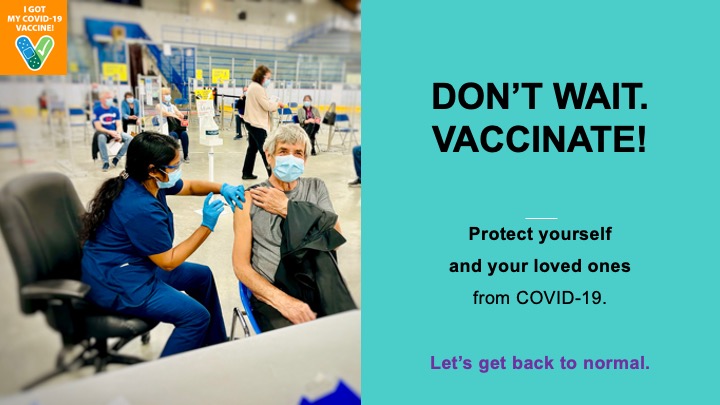

Supplement: Supplementary file 1 [file Data_Sheet_1.docx]
